# Supplementary figures and images for: An antisense oligonucleotide-based strategy to ameliorate cognitive dysfunction in the 22q11.2 Deletion Syndrome
Source: eLife. 2025 May 27;13:RP103328. doi: 10.7554/eLife.103328 (PMC12113277; doi:10.7554/eLife.103328)

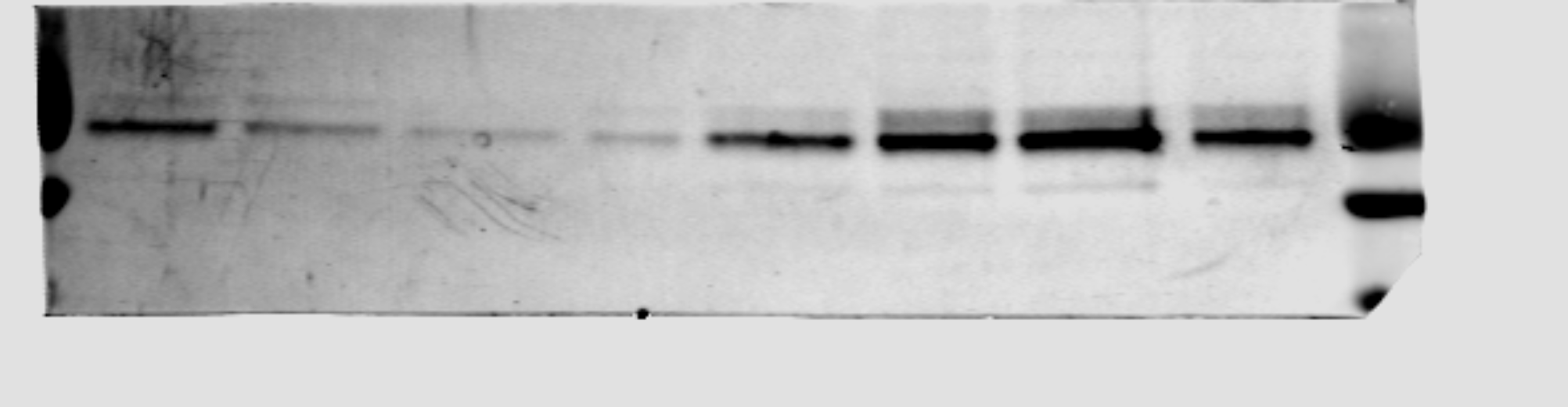

Supplement: Figure 1—source data 2. [file elife-103328-fig1-data2.zip › Figure 1-source data 2/Fig_1G_EMC10.tif]

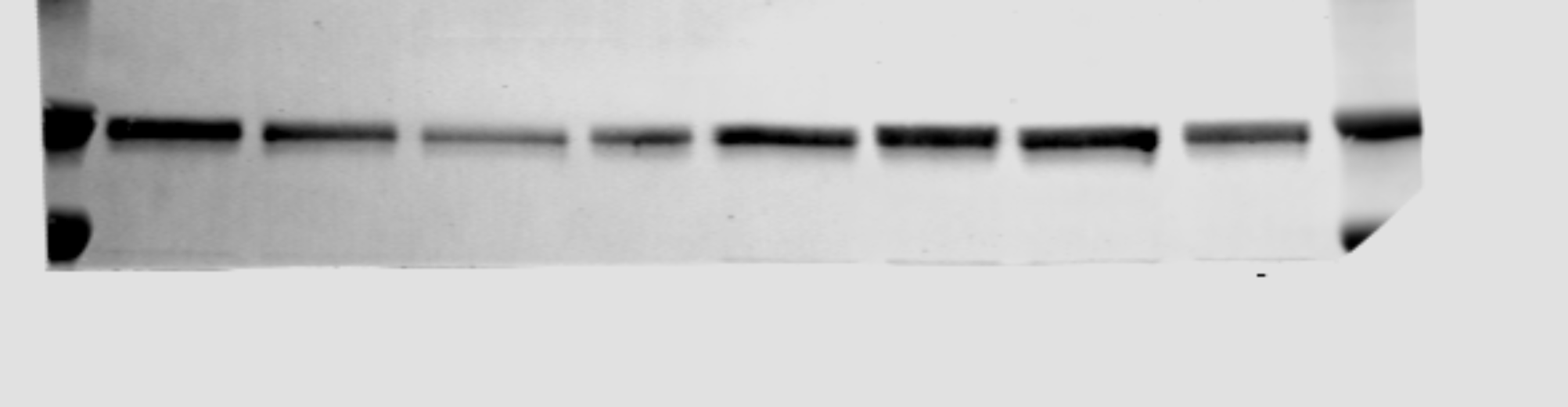

Supplement: Figure 1—source data 2. [file elife-103328-fig1-data2.zip › Figure 1-source data 2/Fig_1G_TUBULIN.tif]

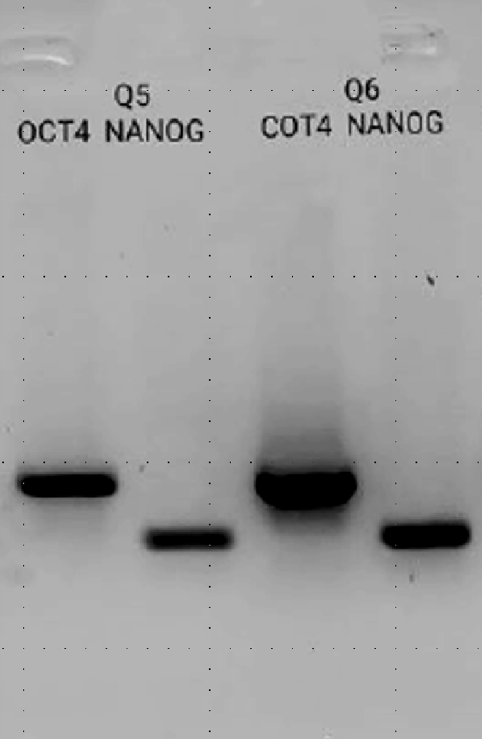

Supplement: Figure 1—figure supplement 1—source data 2. [file elife-103328-fig1-figsupp1-data2.zip › Figure 1-figure supplement 1-source data 2/Fig_1_fig_supp_1D_NANOG_OCT4.tif]

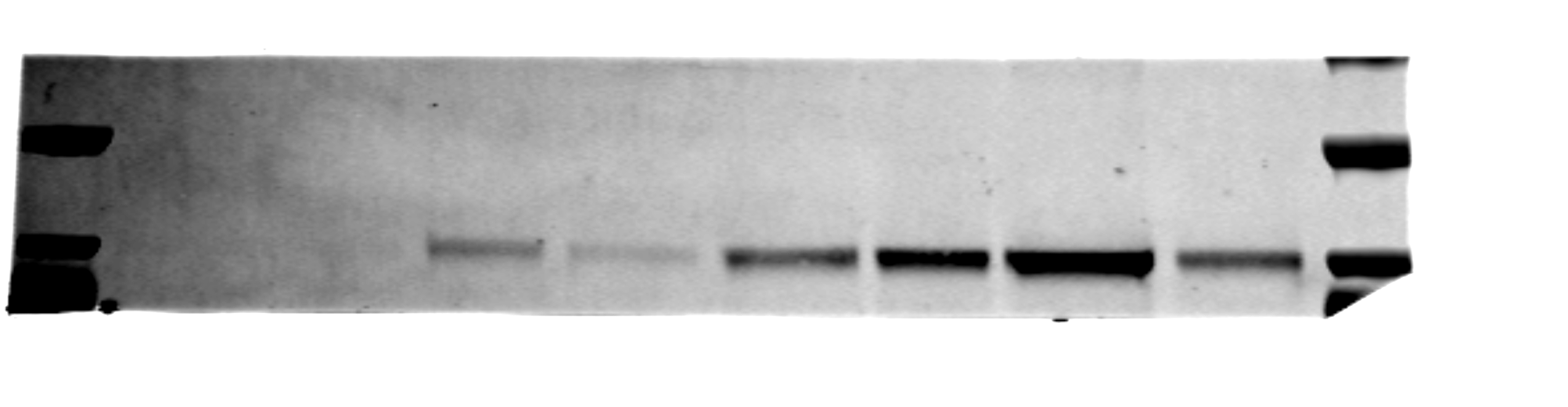

Supplement: Figure 1—figure supplement 4—source data 2. [file elife-103328-fig1-figsupp4-data2.zip › Figure 1-figure supplement 4-source data 2/Fig1-fig_supp_4C_DGCR8.tif]

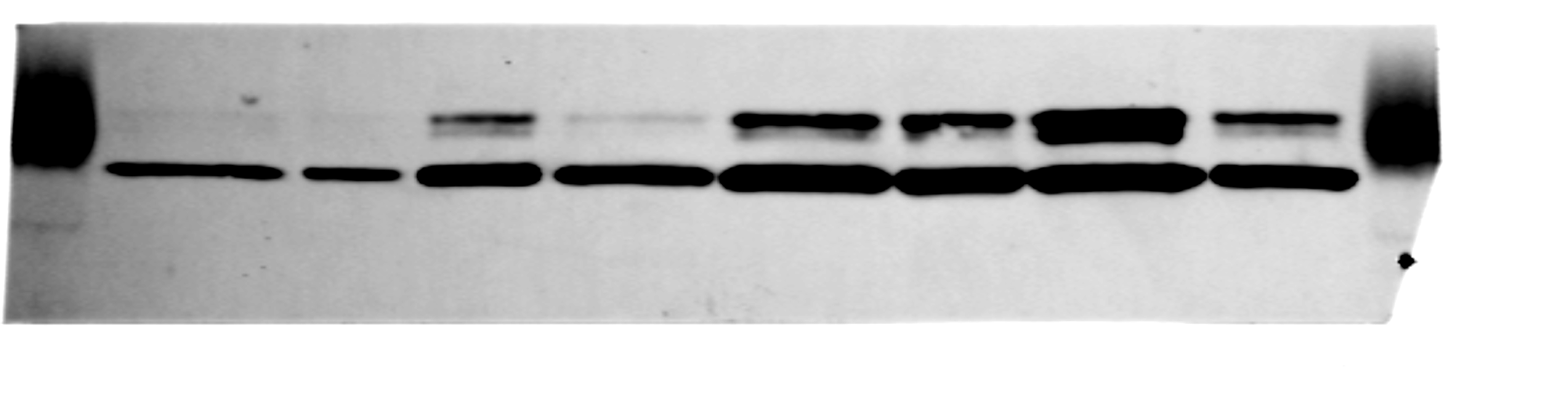

Supplement: Figure 1—figure supplement 4—source data 2. [file elife-103328-fig1-figsupp4-data2.zip › Figure 1-figure supplement 4-source data 2/Fig1-fig_supp_4C_RANBP1.tif]

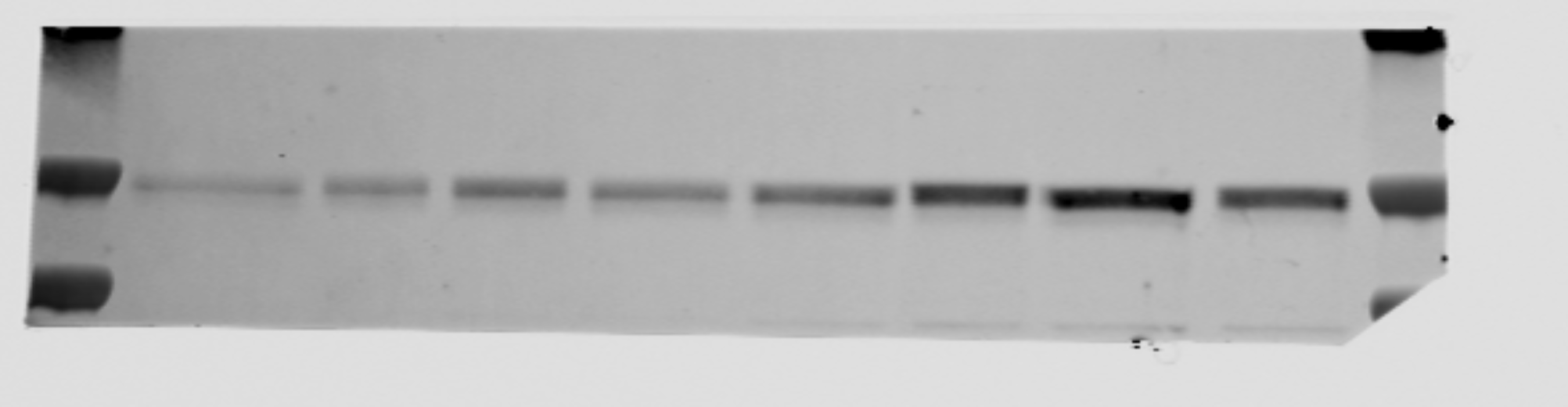

Supplement: Figure 1—figure supplement 4—source data 2. [file elife-103328-fig1-figsupp4-data2.zip › Figure 1-figure supplement 4-source data 2/Fig1-fig_supp_4C_TUBULIN.tif]

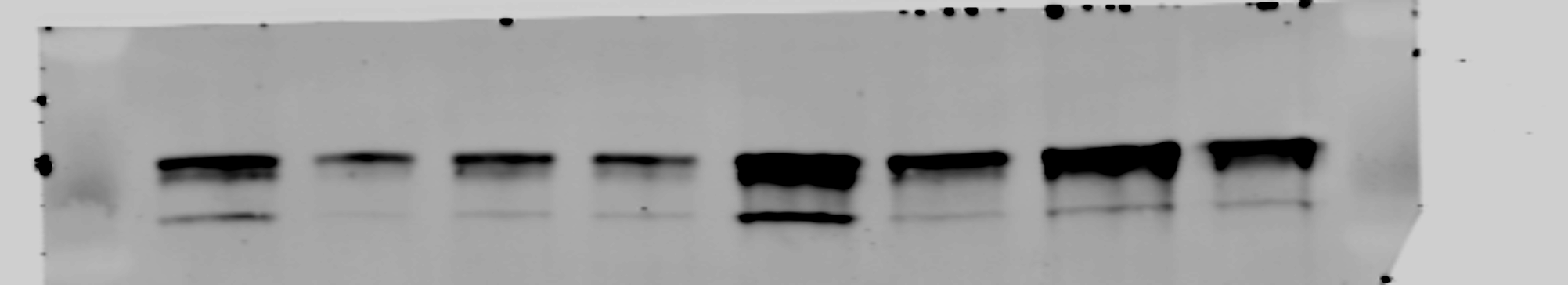

Supplement: Figure 3—figure supplement 1—source data 2. [file elife-103328-fig3-figsupp1-data2.zip › Figure 3-figure supplement 1-source data 2/Fig3-fig-supp_1C_RANBP1.tif]

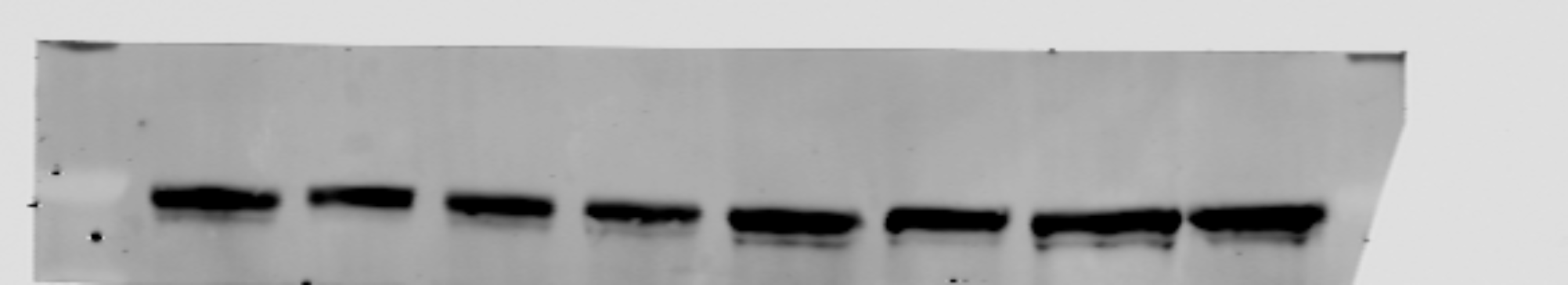

Supplement: Figure 3—figure supplement 1—source data 2. [file elife-103328-fig3-figsupp1-data2.zip › Figure 3-figure supplement 1-source data 2/Fig3-fig-supp_1C_TUBULIN.tif]

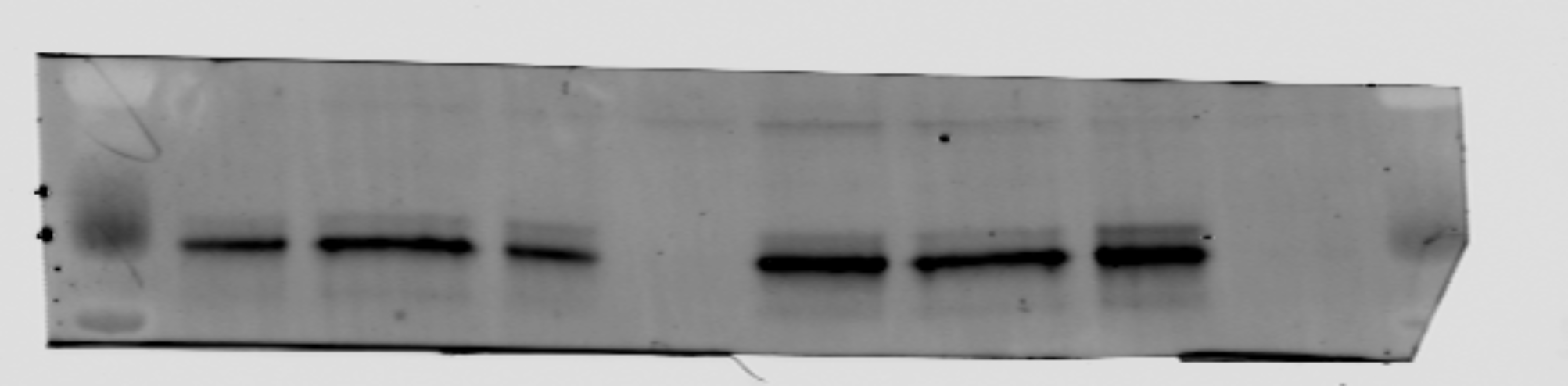

Supplement: Figure 3—figure supplement 1—source data 2. [file elife-103328-fig3-figsupp1-data2.zip › Figure 3-figure supplement 1-source data 2/Fig3-fig-supp_1G_EMC10.tif]

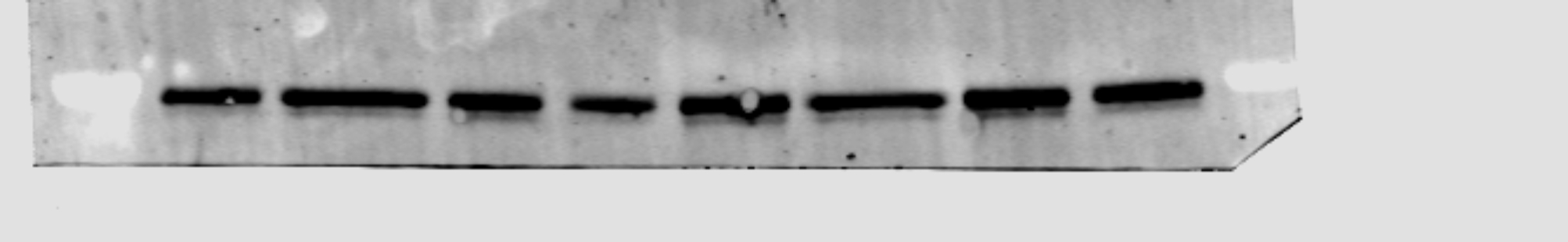

Supplement: Figure 3—figure supplement 1—source data 2. [file elife-103328-fig3-figsupp1-data2.zip › Figure 3-figure supplement 1-source data 2/Fig3-fig-supp_1G_TUBULIN.tif]

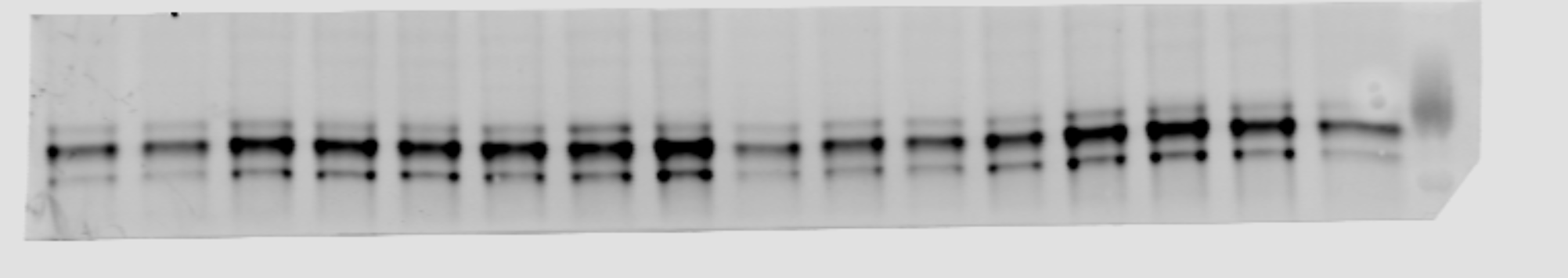

Supplement: Figure 4—figure supplement 2—source data 2. [file elife-103328-fig4-figsupp2-data2.zip › Figure 4-figure supplement 2-source data 2/Fig4-fig-supp_2C_Emc10.tif]

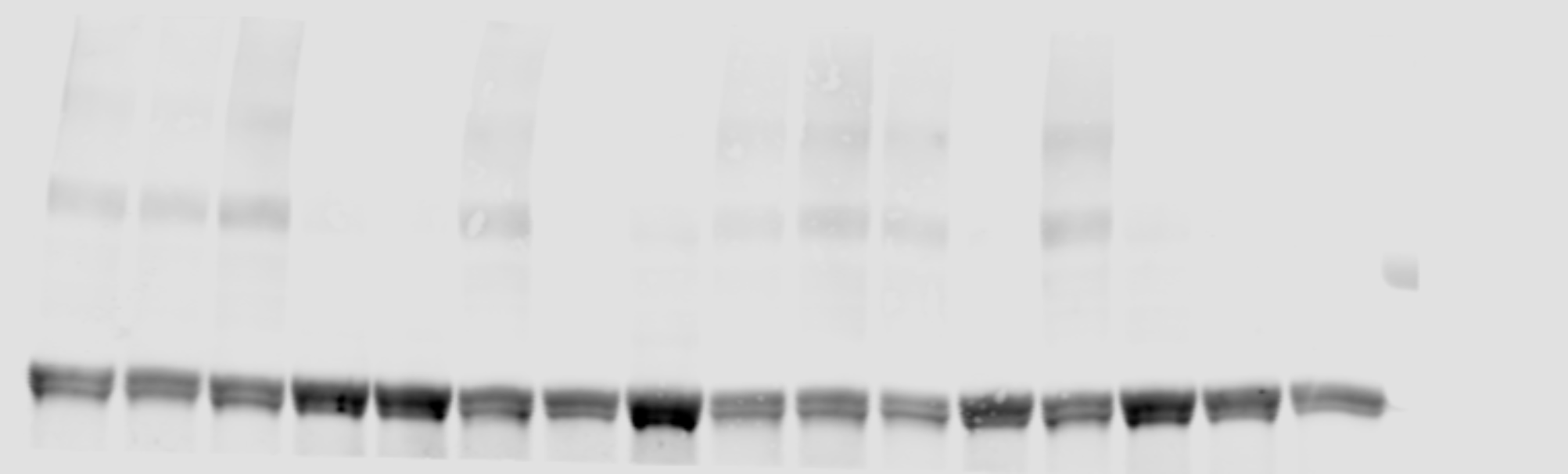

Supplement: Figure 4—figure supplement 2—source data 2. [file elife-103328-fig4-figsupp2-data2.zip › Figure 4-figure supplement 2-source data 2/Fig4-fig-supp_2C_Tubulin.tif]

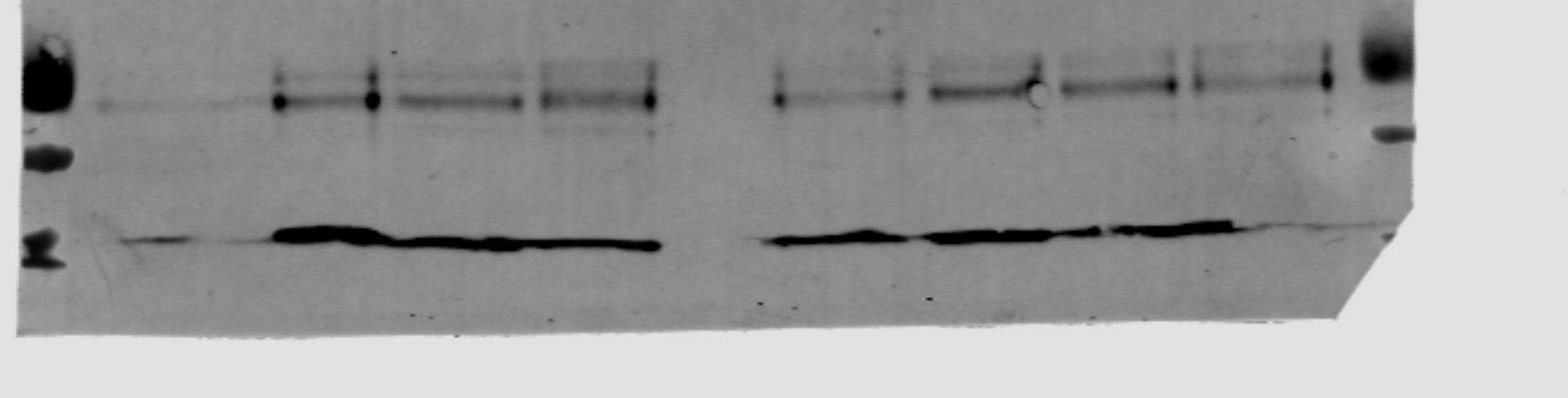

Supplement: Figure 4—figure supplement 3—source data 2. [file elife-103328-fig4-figsupp3-data2.zip › Figure 4-figure supplement 3-source data 2/Fig4-fig-supp_3D-left_Emc10.tif]

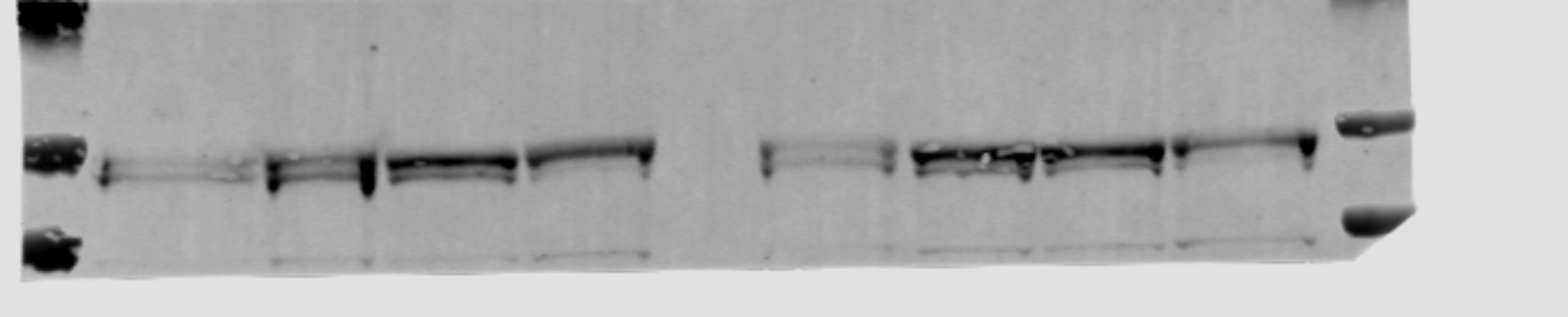

Supplement: Figure 4—figure supplement 3—source data 2. [file elife-103328-fig4-figsupp3-data2.zip › Figure 4-figure supplement 3-source data 2/Fig4-fig-supp_3D-left_Tubulin.tif]

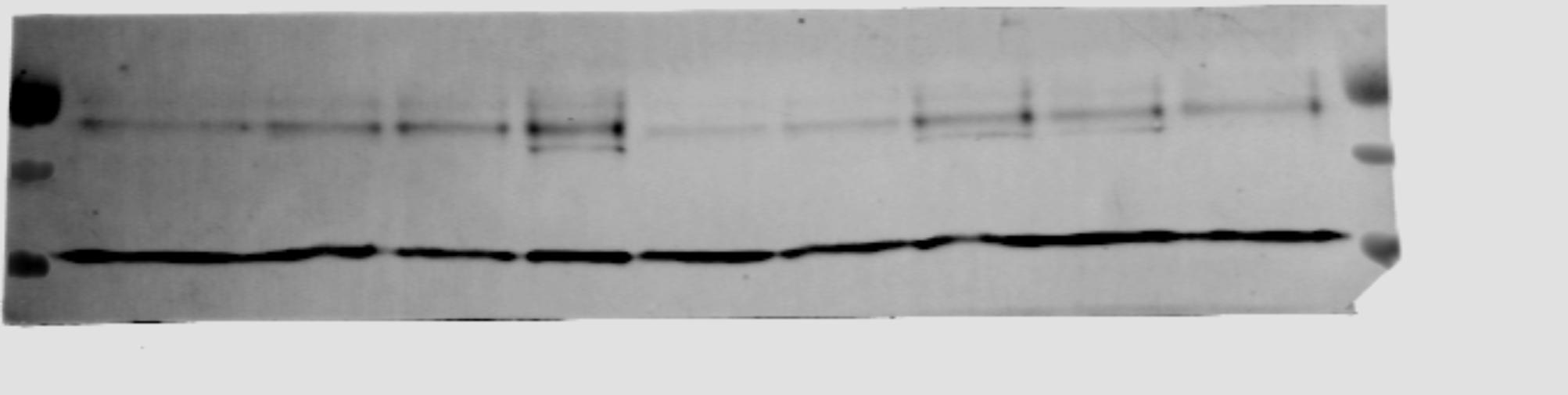

Supplement: Figure 4—figure supplement 3—source data 2. [file elife-103328-fig4-figsupp3-data2.zip › Figure 4-figure supplement 3-source data 2/Fig4-fig-supp_3D-right_Emc10.tif]

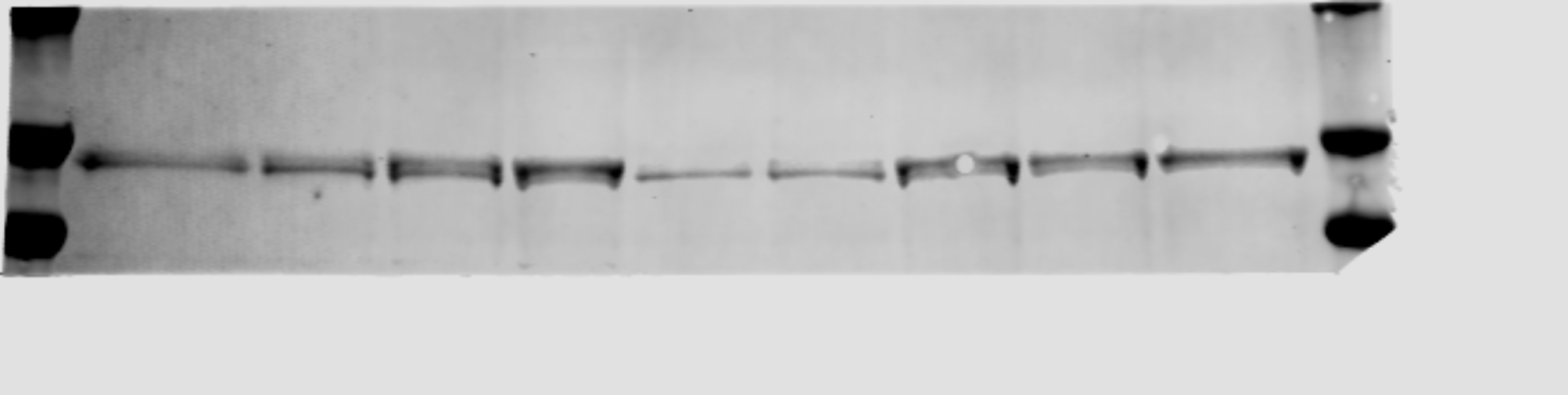

Supplement: Figure 4—figure supplement 3—source data 2. [file elife-103328-fig4-figsupp3-data2.zip › Figure 4-figure supplement 3-source data 2/Fig4-fig-supp_3D-right_Tubulin.tif]
