# Supplementary material for: An antisense oligonucleotide-based strategy to ameliorate cognitive dysfunction in the 22q11.2 Deletion Syndrome
Source: eLife. 2025 May 27;13:RP103328. doi: 10.7554/eLife.103328 (PMC12113277; doi:10.7554/eLife.103328)
Supplement: Figure 1—figure supplement 1—source data 1. [file elife-103328-fig1-figsupp1-data1.pdf]

D

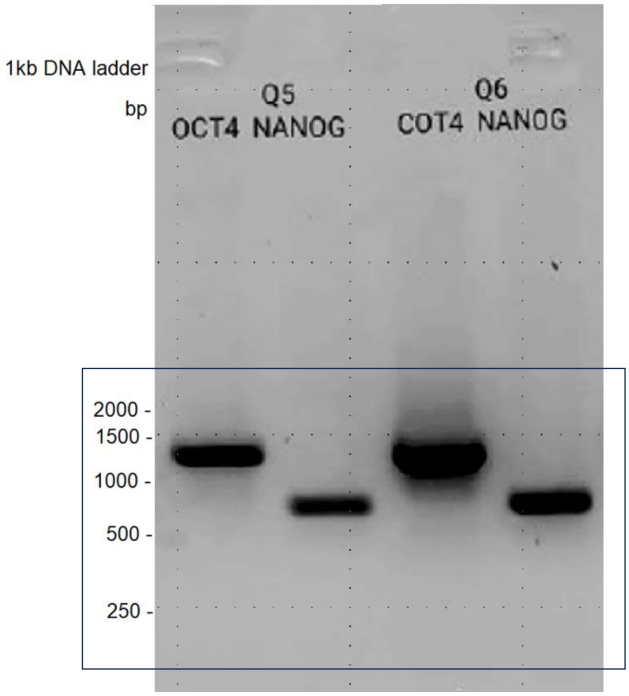

Figure 1-figure supplement 1-source data 1. Original gel corresponding to Figure 1-figure supplement 1D, with relevant bands indicated. Boxed samples are shown in Figure 1-figure supplement panel 1D. The presented gel shows the result of the qRT-PCR assays of embryonic stem cell markers *OCT4/POU5F1* and *NANOG*. The gel shows that they are highly expressed in both hiPSC lines (Q5 Ctrl and Q6 22q11.2).
