# Supplementary material for: An antisense oligonucleotide-based strategy to ameliorate cognitive dysfunction in the 22q11.2 Deletion Syndrome
Source: eLife. 2025 May 27;13:RP103328. doi: 10.7554/eLife.103328 (PMC12113277; doi:10.7554/eLife.103328)
Supplement: Figure 1—figure supplement 4—source data 1. [file elife-103328-fig1-figsupp4-data1.pdf]

C

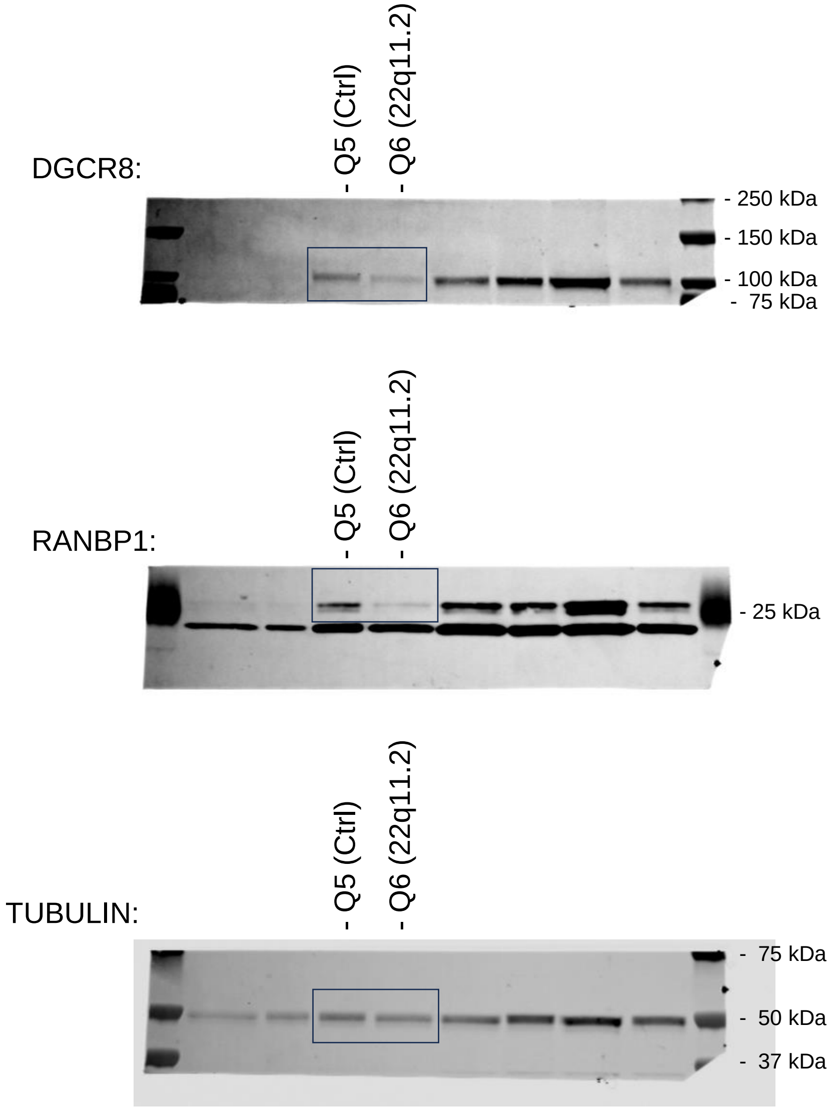

Figure 1-figure supplement 4-source data 1. Original membranes corresponding to Figure 1-figure supplement 4C, with relevant bands and loading controls indicated. Boxed samples are shown in Figure 1-figure supplement panel 4C. For all membranes the Precision Plus Protein Dual Color Standards (Bio-Rad, Hercules, CA, USA) molecular weight marker was used. Western blot analysis showing reduced amount of DGCR8 and RANBP1 protein in Q6 (22q11.2) line derived cortical neurons at day 8 of differentiation. Tubulin was probed as a loading control.
