# Supplementary material for: An antisense oligonucleotide-based strategy to ameliorate cognitive dysfunction in the 22q11.2 Deletion Syndrome
Source: eLife. 2025 May 27;13:RP103328. doi: 10.7554/eLife.103328 (PMC12113277; doi:10.7554/eLife.103328)
Supplement: Figure 3—figure supplement 1—source data 1. [file elife-103328-fig3-figsupp1-data1.pdf]

C

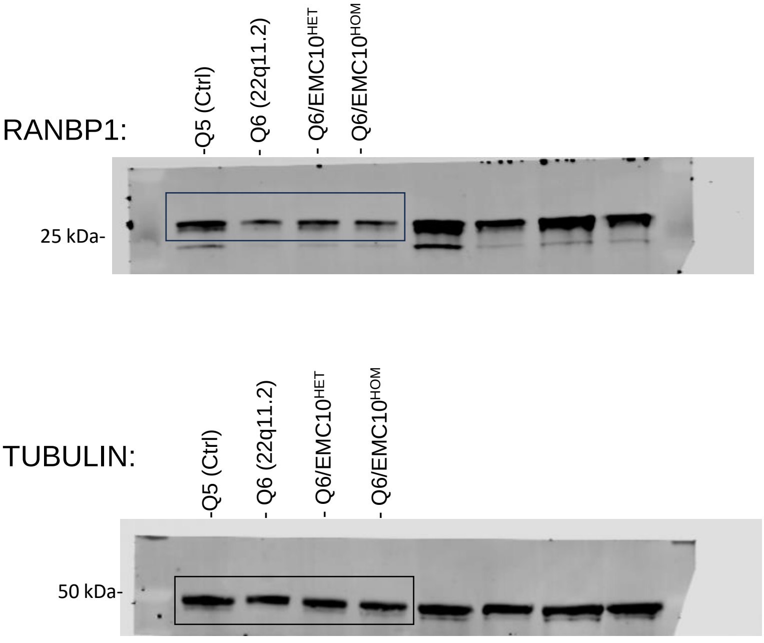

G

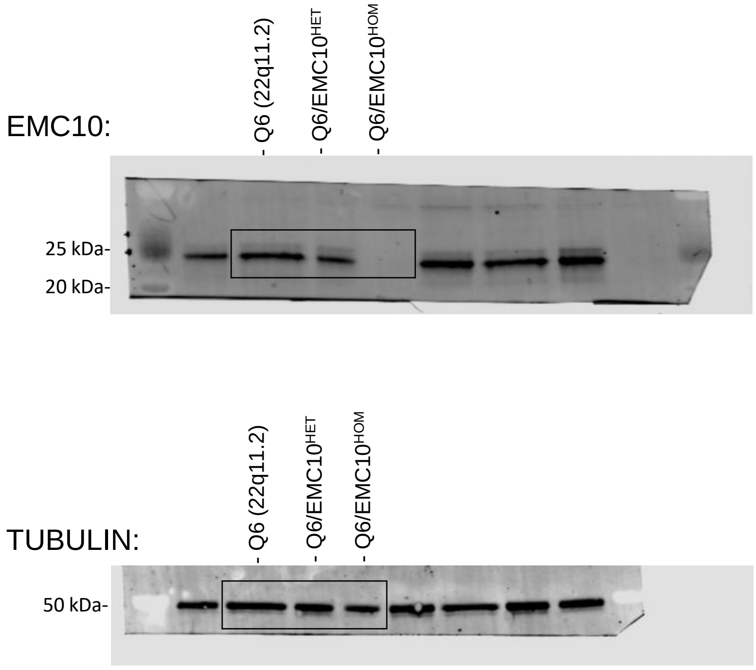

Figure 3-figure supplement 1-source data 1. Original membranes corresponding to Figure 3-figure supplement 1C and G, with relevant bands and loading controls indicated. Boxed samples are shown in Figure 3-figure supplement panels 1C and G. For all membranes the Precision Plus Protein Dual Color Standards (Bio-Rad, Hercules, CA, USA) molecular weight marker was used. (**C**) Western blot analysis confirmed reduction of RANBP1 protein levels in Q6, Q6/EMC10^{HET} and Q6/EMC10^{HOM} lines. (**G**) Western blot analysis showing reduction (Q6/EMC10^{HET}) or elimination (Q6/EMC10^{HOM}) of EMC10 protein levels in the *EMC10* LoF mutant hiPSC lines. Tubulin was probed as a loading control.
