# Supplementary material for: An antisense oligonucleotide-based strategy to ameliorate cognitive dysfunction in the 22q11.2 Deletion Syndrome
Source: eLife. 2025 May 27;13:RP103328. doi: 10.7554/eLife.103328 (PMC12113277; doi:10.7554/eLife.103328)
Supplement: Figure 4—figure supplement 2—source data 1. [file elife-103328-fig4-figsupp2-data1.pdf]

C

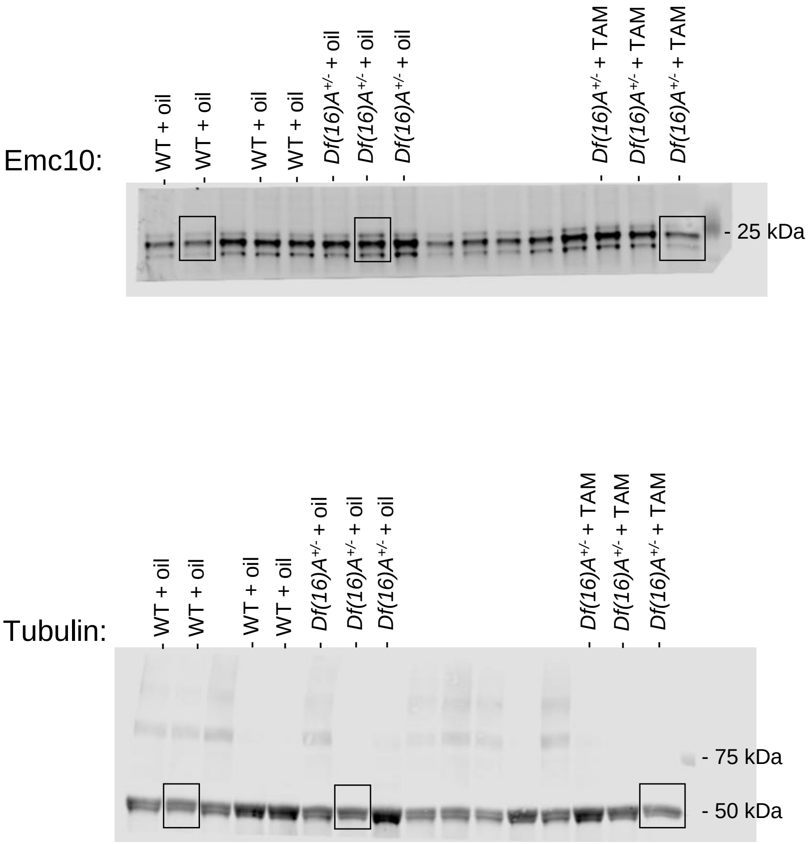

Figure 4-figure supplement 2-source data 1. Original membranes corresponding to Figure 4-figure supplement 2C, with relevant bands and loading controls indicated that were used for quantification. Boxed samples are shown in Figure 4-figure supplement 2 panel C (right). For all membranes the Precision Plus Protein Dual Color Standards (Bio-Rad, Hercules, CA, USA) molecular weight marker was used. Emc10 protein levels in PFC of WT and *Df(16)A*$^{+/-}$ mice following adult TAM treatment are shown. Emc10 protein level is reduced in TAM-treated *Df(16)A*$^{+/-}$ compared to corn oil-treated *Df(16)A*$^{+/-}$ mice. Tubulin was used as loading control.
