# Supplementary material for: An antisense oligonucleotide-based strategy to ameliorate cognitive dysfunction in the 22q11.2 Deletion Syndrome
Source: eLife. 2025 May 27;13:RP103328. doi: 10.7554/eLife.103328 (PMC12113277; doi:10.7554/eLife.103328)
Supplement: Figure 4—figure supplement 3—source data 1. [file elife-103328-fig4-figsupp3-data1.pdf]

D

Emc10 (left HPC):

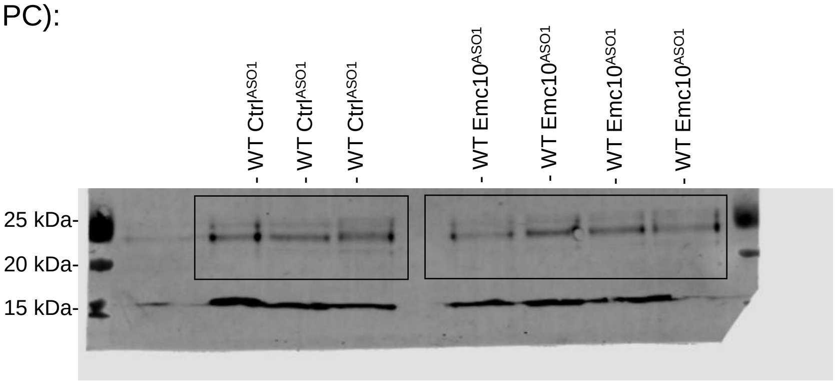

Tubulin (left HPC):

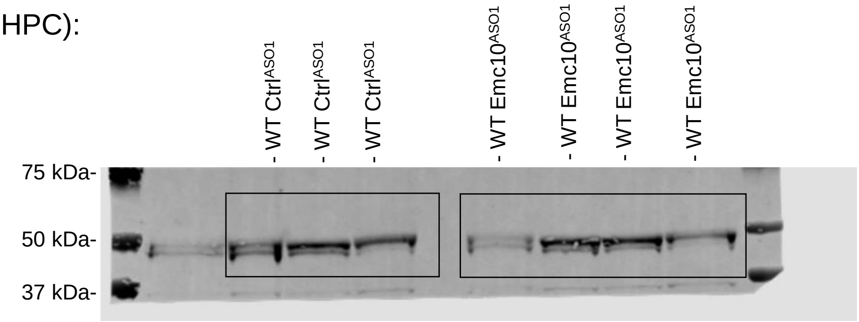

Emc10 (right HPC):

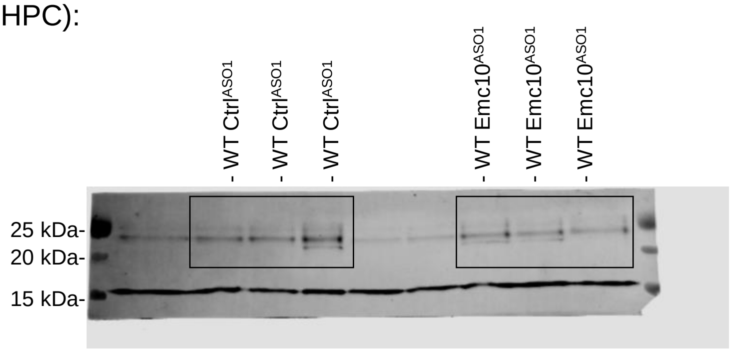

Tubulin (right HPC):

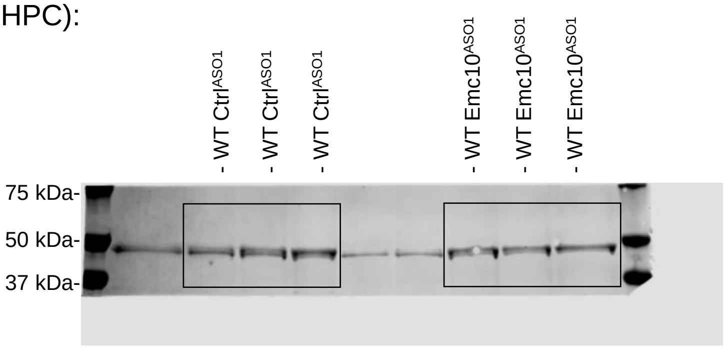

Figure 4-figure supplement 3-source data 1. Original membranes corresponding to Figure 4-figure supplement 3D, left and right HPC, with relevant bands and loading controls indicated. Boxed samples were used for quantification shown in Figure 4-figure supplement panel 3D. For all membranes the Precision Plus Protein Dual Color Standards (Bio-Rad, Hercules, CA, USA) molecular weight marker was used. Western blot shows reduction of Emc10 protein expression in the left and right HPC after ASO treatment. Tubulin was used as loading control.
